# Anaphylaxis associated with folic acid: domestic case review

**DOI:** 10.1186/1710-1492-10-S2-A23

**Published:** 2014-12-18

**Authors:** Pauline E Kerr, David Cunningham

**Affiliations:** 1 Marketed Health Products Directorate, Health Products and Food Branch, Health Canada, Ottawa, Ontario, Canada, K1A 0K9

## Background

Synthetic folic acid has been shown to cause serious, potentially life-threatening IgE mediated anaphylaxis [1-3]. Synthetic folic acid does not occur naturally and is distinct from naturally occurring folic acid [1,4,5].

## Methods

A search of the Canada Vigilance Database from April 1, 2008 to August 31, 2013 identified 15 cases of anaphylaxis and 16 reports of serious non-anaphylactic allergic reactions.

## Results

All 15 cases of anaphylaxis involved multi ingredient products with NHP doses (up to and including 1 mg). Fourteen cases were female (93%). One case of anaphylaxis and 3 cases of serious allergic reaction involved women taking prenatal supplements, two of which reported spontaneous abortion. There was insufficient case information available to assess any causal association between the allergic reaction and the fetal loss. Three medically important cases involved pharmaceutical doses of folic acid (5 mg), one of which was a pediatric case involving a multi ingredient intravenous total parental nutrition product (Multi-12/K1 pediatric) containing folic acid.

Confirmatory allergy tests for folic acid were not available for any of the cases. None of the reports noted a known diagnosis of folic acid allergy.

## Conclusions

Folic acid allergy appears to be rare and would not be expected to be well known among health providers or consumers. Increasing awareness amongst health providers regarding folic acid allergy may improve the identification and counselling of patients with this allergy.
